# *Coxiella burnetii* in slaughterhouses in Brazil: A public health concern

**DOI:** 10.1371/journal.pone.0241246

**Published:** 2020-10-30

**Authors:** Mateus de Souza Ribeiro Mioni, Francisco Borges Costa, Bruna Letícia Devidé Ribeiro, Wanderson Sirley Reis Teixeira, Vanessa Cristina Pelicia, Marcelo Bahia Labruna, Élodie Rousset, Karim Sidi-Boumedine, Richard Thiéry, Jane Megid

**Affiliations:** 1 Departamento de Higiene Veterinária e Saúde Pública, Universidade Estadual Paulista “Júlio de Mesquita Filho”, Botucatu, São Paulo, Brazil; 2 Departamento de Patologia, Universidade Estadual do Maranhão, São Luís, Maranhão, Brazil; 3 Instituto de Química, Universidade de São Paulo, São Paulo, São Paulo, Brazil; 4 Departamento de Medicina Veterinária Preventiva e Saúde Animal, Universidade de São Paulo, São Paulo, São Paulo, Brazil; 5 Anses, French Agency for Food, Environmental and Occupational Health Safety, Sophia Antipolis laboratory, Animal Q Fever Unit, Sophia Antipolis, France; University of Lincoln, UNITED KINGDOM

## Abstract

Q fever is an important zoonosis, yet it is often neglected and can present large outbreaks, as observed in the Netherlands. In the past few years, cases of Q fever have been described in Brazil; however, the epidemiological situation of Q fever in ruminants, the main reservoir of the pathogen, is unknown in this country. Our study aimed to estimate the prevalence of *C*. *burnetii* in cattle sent to slaughterhouses using an immunofluorescence assay (IFA) and quantitative real-time PCR (qPCR). From 1515 cattle serum samples collected from nine slaughterhouses, 23.8% (360/1515) were serologically positive by IFA (cutoff titer>1:64), indicating past or recent exposure to *C*. *burnetii* infection. Among the 54 cities sampled during the study, 83.3% (45/54) had at least one seropositive animal. Subsequently, all seropositive samples were submitted to qPCR for *C*. *burnetii* DNA, and 12.2% (44/360) of the sera were qPCR positive, which indicates bacteremia and suggests active or recent infection. The results highlight the risk for abattoir workers that results from exposure to contaminated aerosols produced during slaughter procedures. Moreover, the heat maps that were construction from the positive samples demonstrate the widespread distribution of *C*. *burnetii* in the State of São Paulo, Brazil and denotes the need for surveillance and preventive measures to reduce the prevalence in cattle.

## Introduction

*Coxiella burnetii* is the causative agent of Q fever in humans and animals [1, 2], and this condition is distributed throughout the world, with the exception of New Zealand [1, 3]. It is an obligatory intracellular, gram-negative bacterium that can persist in the environment for long periods, due to its highly infectious spore-like form [3].

In humans, Q fever is mainly asymptomatic. However, an acute form with flu-like syndromes, acute hepatitis, pneumonia, and a chronic and more severe form with persistent focalized infection, such as endocarditis or vascular infections, are also possible [2, 4]. *C*. *burnetii* is mainly transmitted by the inhalation of aerosol particles in the vicinity of infected animals, their reproductive tissues, or other animal products [1]. However, people who live far from livestock can also acquire the disease through the inhalation of particles transported by wind [5, 6]. Additionally, *C*. *burnetii* can be transmitted orally through the consumption of milk and dairy products, although this route has a minor importance in the epidemiology of the disease [1, 7]. Moreover, despite the fact that *C*. *burnetii* has been detected in ticks the possibility of transmission to humans through the bite of these arthropods is low. Nevertheless, ticks may play a role in transmission to animals due to ecological features (e.g. host preference and bite rate) [8].

Coxiellosis is most often latent in animals, without clinical manifestation, but may cause bacteria sheeding into the environment via feces or parturition products [1]. *C*. *burnetii* can infect a diverse group of species that range from arthropods and cold blooded animals to birds and mammals [1, 3, 8], and domestic ruminants are considered the main reservoirs for human disease [1, 9]. In cows, ewes, and goats, the disease is associated with late abortion. Reproductive disorders, such as metritis and infertility, have also been associated with the disease [1].

The worldwide prevalence of coxiellosis is somewhat higher in cattle than in small ruminants [10], although, in European countries, goats, followed by ewes, are the species with the highest infection rates [11, 12]. In Brazil, only a few epidemiological surveys have aimed to establish seroprevalence in animals. In 1955, Valle et al. [13] found 14% (24/171) positivity in dairy cows. Later, Riemann et al.[14] reported a seroprevalence of 29% (45/156) in beef cattle. Recently, a survey using a commercial ELISA found 55.1% (172/312) seropositivity in a goat herd with reproductive disorders [15], and Mioni et al. [16] reported the presence of *C*. *burnetii* in dairy cattle and their fetuses in the state of Goiás [17].

Lipopolysaccharide (LPS) is the main *C*. *burnetii* antigen and is found in two different phases on the bacterium surface. The phase I antigen is the complete form of LPS and is linked to the virulence. The phase II antigen is the truncated form that is obtained after several passages in cell culture, and it is the result of gene deletion and consequent loss of sugars from LPS [2, 9]. Serological antigens are based on these two antigenic forms of *C*. *burnetii* [1, 2]. Phase II is imunodominant and is inserted in phase I LPS. For this reason, antibodies against phase II are always present during the course of infection, and this feature is important for epidemiological studies where the detection of phase II antibodies is preferable [18].

In humans, the serodiagnostic is based on the different titers of IgG to phase I and phase II antigens. Antibodies against phase II are the first to arise in primo infections; and for this reason, such antibodies are linked to acute Q fever, whereas the increase in the antibody response to phase I (IgG≥1:800 when using immunofluorescence assay–IFA) is related to persistent focalize infections [2]. In animals, although the antibody response to the infection has the same pattern, the interpretation of the antibody response to phase I and phase II antigens has not been validated for diagnosing acute and chronic Q fever [1]. Instead, commercially available kits can detect mixtures of anti-phase I and II antibodies to reflect the total immunoglobulin content in the sera [1].

Moreover, for acute Q fever in humans, the use of real-time PCR (qPCR) in the early stages of the disease is necessary due to the lack of antibodies in the first two weeks of symptoms and evidentiate bacteremia [2, 19]. This approach is essential for the diagnosis of clinical manifestations after Q fever primo-infection and the identification of new cases during outbreaks, in association with IgM rates [19, 20]. However, only a few studies have reported molecular diagnoses using serum samples [21–25]. Moreover, due to a lack of data, it is difficult to establish its significance in animal disease.

Q fever is considered an occupational hazard for farmers, veterinarians, and abattoir workers [26]. Since its description in Australia in 1937, epidemic Q fever was predominantly associated with abattoirs in that country, until 1980. In some regions, 92% of cases of the disease were among slaughterhouse workers [27, 28]. In France, one slaughterhouse was the source of a Q fever outbreak that affected 29 people [29]. In Brazil, a survey reported 29% (41/144) of seroprevalence for *C*. *burnetii* among cattle slaughterhouse workers, and the positivity was higher for those who worked in corrals (40%) and the killing floor (36%) [14]. In 2015, an outbreak related to cattle slaughterhouse workers was reported in the city of Barbosa (21°160″S 49°5654″W), which is located in the state of São Paulo [30]. Apart from slaughterhouse scenarios, human Q fever in Brazil has been confirmed in patients with endocarditis [31–33], patients suspected of having dengue [34, 35], and a family cluster case [36]. Additionally, the most recently reported case of Q fever involved six cadets after a military training where they had contact with a goat and capybaras [37, 38]. The state of Rio de Janeiro is the region of Brazil with the most reports of Q fever.

Although the risk of human outbreaks is usually linked to small ruminants; in Brazil, the Q fever can be due to a different scenario, since the country is a primary producer of bovine meat, with a herd of more than 214 million animals. In comparison, the flocks of small ruminants combined are limited to 28 million animals [39].

The public health link to Q fever in Brazil is unknown, as is the epidemiological status regarding the prevalence of *C*. *burnetii* in reservoirs and the risk of acquiring Q fever during the slaughter of animals. Therefore, this study aimed to establish the prevalence of *C*. *burnetii* in beef cattle in the State of São Paulo, Brazil, using serological and molecular diagnostic methods.

## Materials and methods

The Ethics Committee on Animal Use (CEUA-FMVZ-UNESP/Botucatu, State of Sao Paulo, Brazil) approved this study (protocol numbers 141/2013 and 0203/2016). CEUA-FMVZ-UNESP/Botucatu is registered in the National Council for Animal Control and Experimentation (CONCEA), under registration number CIAEP/CONCEA 01.0115.2014. The CONCEA is regulated by LAW No. 11.794, October 8th, 2008, which establishes procedures for the scientific use of animals. Moreover, the CONCEA is responsible for the periodic review of standards for use and care of animals for teaching or scientific research proposes, following the international conventions of which Brazil is a participant.

The Ministry of Agriculture, Livestock, and Food Supply (MAPA), the Secretariat of Agriculture and Supply of the State of São Paulo, and the municipal authorities of the cities involved, as well as those responsible for each establishment, provided permission to sample animals in slaughterhouses.

### Samples

Serum samples were obtained from nine slaughterhouses in the state of São Paulo, Brazil, from October 2014 to March 2015. To calculate the sample size, we used an Openepi proportion test [40]. To estimate the number of bovines to be tested through IFA, a slaughter population of 3.5 million bovines per year in the State of São Paulo was estimated [41]. As the real situation of the disease is unknown, we used an apparent coxiellosis prevalence of 50%, with 3% precision and a confidence interval of 95%. Using these parameters, the minimum sample size needed to estimate the prevalence in the slaughter population was 1066 samples. To avoid sample bias, we collected 1515 cattle (*Bos taurus* and *Bos indicus*) samples, which were divided among the nine slaughterhouses in the state of São Paulo, with a minimum of 157 samples per facility. All facilities in the experiment were sampled only after authorization by the authority of the inspection system of the establishment. Animals were sampled without breed and sex discrimination. Blood samples were collected at the bleeding step after desensitization and vessel sectioning. Blood samples were collected in tubes (BD Vacutainer Serum Plus Blood collection tubes with a clot activator, 10 mL; BD, Franklin Lakes, NJ), and serum was obtained by centrifugation (2,000g /10 min) within 12 h. All serum samples were stored at– 80 °C until the time of analysis.

### Immunofluorescence assay

Serum samples were defrosted, diluted two-fold, and analyzed by an IFA, according to Horta *et al*. [42], with a cutoff of 1:64. For the IFA, we used slides that contained a mixture of phase I and phase II *C*. *burnetii* antigens, and the *C*. *burnetii* were purified after production on Vero cells, to detect total immunoglobulin (IgM and IgG). The in-house antigen was produced from the Argentinian *C*. *burnetii* strain At12, which was isolated from *Amblyomma tigrinum* [43]. Positive samples were subjected to real-time PCR for the detection of *C*. *burnetii* in the bloodstream.

For serological analysis, all slides used during the assay were assembled as follows: 20 μL of serum samples were placed in the wells that were previously prepared with the antigen and incubated for 30 min at 37 °C. The slides were then washed with washing buffer (WB) and placed for 10 min in a vat that contained WB solution, and this operation was repeated twice. Subsequently, the slides were dried at 37 °C for 10 min. The material was organized in a humid chamber, and 20 μL of the bovine conjugate was added and incubated for 30 min at 37°C. After incubation, slides were washed with WB and deposited in a vat that contained WB solution and 1.5 mL of Evans solution, for 10 min (step repeated twice). The slides were dried and read under a fluorescence microscope. In each slide, positive and negative sera were used as controls. Sera that showed reactivity following a 1:64 dilution were considered positive.

### DNA extraction

The extraction of serum samples was carried out with a DNeasy Blood and Tissue Kit (Qiagen, Hilden, Germany) according to the manufacturer’s protocol.

### Real-time PCR

All samples that showed a positive IFA reaction (n = 360) were screened in Brazil through real-time PCR using a melt analysis protocol. For quantification, qPCR was performed using specific probes for *C*. *burnetii* as well as reference materials prepared from the Nine Mile strain. The procedure was performed at the French Agency for Food, Environmental, and Occupational Health Safety (ANSES, Sophia Antipolis, France), which is mandated as the French National Reference Laboratory and a World Organization for Animal Health (O.I.E.) reference laboratory for Q fever.

The melt-analysis protocol was performed according to Vaidya *et al*. [44] with primers Trans 3 (5'-GTAACGATGCGCAGGCGAT-3') and Trans 4 (5'-CCACCGCTTCGCTCGCTA-3'), as previously described [45] for the detection of the *IS1111a* element. The qPCR assay was performed in a Real-Time Applied Biosystems Step-One (model ABI 7500 Fast), using the standard cycle. As a positive control, we used DNA extracted from *C*. *burnetii* strain At12, which was isolated from ticks [43]. The positive control dissociation curve was recorded a melting temperature (Tm) of 86.91°C.

For quantifying *C*. *burnetii* in the samples, qPCR was used with specific primers and probes developed by the reference center. Briefly, the PCR reaction was performed in a total volume of 25 μL that contained 320 nM of each forward and reverse primer, 200 nM of the specific probe (FAM-TAMRA), 1X × TaqMan Universal PCR Master Mix (Applied Biosystems), 5.7 μL nuclease-free PCR grade water, and 5 μL of DNA template. As a control, primers (320 nM) targeting the glyceraldehyde-3-phosphate dehydrogenase (GAPDH) gene of ruminants and the VIC-TAMRA probe at a concentration of 200 nM were used. All assays were performed using CFX96^™^ equipment (Bio-Rad Laboratories, Inc.). Cycling conditions were as follows: 50°C for 2 min, 95°C for 10 min, and 40 cycles of 95 / 15 s, 60°C / 1 min. To quantify the samples, in each assay, a standard curve that ranged from 2×10^2^ to 2×10^7^ genome equivalent (GE) bacteria/mL was prepared using a tenfold dilution of purified genomic DNA from a calibrated *C*. *burnetii* Nine Mile strain. The theoretical number of GEs was calculated based on the measured DNA concentration and the length of the *C*. *burnetii* Nine Mile genome sequence (1,995,275 bp) published by Seshadri et al. [46]. As a quality control for the assay, two sentinel samples that contained Nine Mile isolates at a concentration of 10^4^ were used to check the accuracy and reproducibility of the quantification.

### Sequencing

To confirm the detection of *C*. *burnetii*, we sequenced seven positive samples by qPCR. They were analyzed by 1% agarose gel, purified with Agencourt AMPure XP (Beckman Coulter, Inc.), and subjected to automatic sequencing by capillary electrophoresis on an ABI3500 Genetic Analyzer (Applied Biosystems). The nucleotide sequences that were produced were aligned with the reference sequences deposited in GenBank. The PCR product was also confirmed by 1.5% agarose gel electrophoresis with ethidium bromide, to visualize a 243 bp product. Seven sequence analyses that were produced during this survey were deposited in GenBank (accession numbers MK125016, MK125017, MK125018, MK125019, MK125020, MK125021, and MK125022).

### Phylogenetic analysis

To confirm the detection of *C*. *burnetii*, we performed a phylogenic analysis using the online tool phylogeny.fr [47–49] with the seven sequences generated here, sixteen *IS1111a C*. *burnetii* strain sequences from different countries and hosts, and an *IS1111*-like sequence from a Coxiella-like bacteria (CLB) with 90% identity with the insertion element of *C*. *burnetii* strains [50]. All GenBank accession numbers of the sequences used in this study are available in the phylogenetic tree.

### Georeferencing

All animals evaluated were spatially distributed in the cities of origin, sampled during work, and analyzed through thematic density maps with ArcGIS 10.0 [51]. We created three sample distribution thematic maps that showed i) the total number of samples, ii) the number of serologically-positive samples by IFA, and iii) the number of serum samples that were positive in the real-time PCR analysis. All heat maps represent the origin of the bovines that were sampled in the different slaughterhouses.

The true prevalence was calculated considering the sensibility and specificity values that were previously reported to IFA [52] in ruminants. All statistical analyses took into account a confidence interval of 95% and were performed with “epiR” [53] in the R environment [54].

## Results

The apparent and true prevalence observed by IFA was 23.8% (CI95% = 21.7%– 25.9%) (360/1515) and 20.0% (CI95% = 17.8%– 22.3%), respectively. Among seropositive samples, the presence of *C*. *burnetii* DNA was observed by qPCR in 12.2% of the cattle (44/360). When we analyzed the seroprevalence and detection by slaughterhouse, we observed homogenous *C*. *burnetii* positivity, since all facilities had positive samples in both diagnostic methods (Table 1). Moreover, the chi-squared test showed no statistical difference (p>0.05) in seroprevalence among the slaughterhouses.

**Table 1:** Prevalence for each slaughterhouse evaluated during the study by IFA and qPCR.

Slaughterhouse	Samples analyzed (n)	Positives (n)	Prevalence (%)	Samples analyzed (n)	Positives (n)	Positivity (%)
1	170	42	24.7%	42	8	19.0%
2	166	38	22.9%	38	7	18.4%
3	170	29	17.0%	29	3	10.3%
4	170	49	28.8%	49	5	10.2%
5	181	64	35.3%	64	2	3.1%
6	158	27	17.1%	27	15	55.6%
7	162	42	25.9%	42	3	7.1%
8	172	21	12.2%	21	0	0.0%
9	166	48	28.9%	48	1	2.1%
Total	1515	360	23.8%	360	44	12.2%

Quantification of the positive serum samples showed an average of 144 bacteria/mL in the serum, with a minimum of 10 bacteria and a maximum of 814 bacteria/mL. For the average estimation, we used only samples with Ct < 36.

All *IS 1111a* sequences presented 100% identity with the *C*. *burnetii* strains available in GenBank. The phylogenetic analysis confirmed the diagnosis of *C*. *burnetii* (S1 Fig), as all *C*. *burnetii* strains were clustered together, and the *IS 1111-like* sequence of the CLB detected on a tick was placed in a different branch.

**Fig 1:**
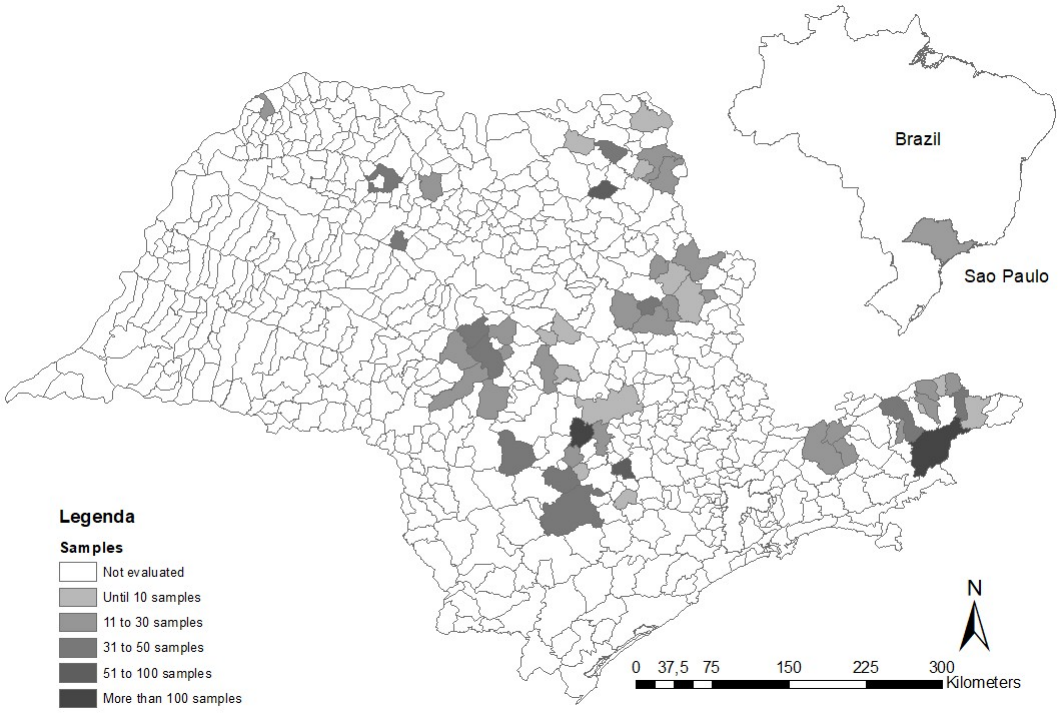
Distribution of collected serum samples by municipalities.

**Fig 2:**
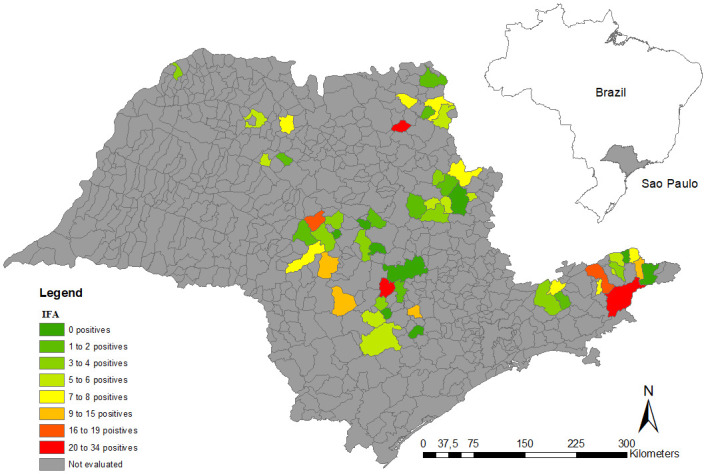
Distribution of seropositive *C*. *burnetii* animals based on the immunofluorescence assay (IFA).

**Fig 3:**
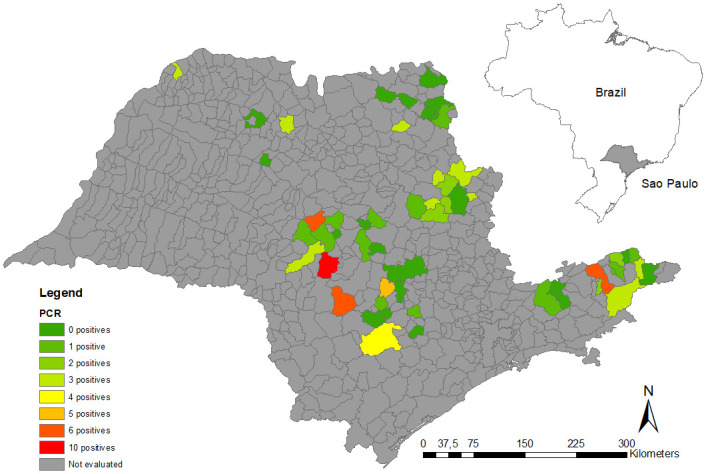
Detection of *Coxiella burnetii* DNA in cattle from different municipalities.

Regarding the positive sample distribution, density maps were constructed, and using the herd location of the slaughtered animals allowed revealed that *C*. *burnetii* circulates across the state of São Paulo, since we observed positive serological and molecular diagnoses in all sampled regions (Figs 1–3). Using slaughterhouses as a tool for Q fever surveillance, we were able to sample animals derived from 54 cities across the state of São Paulo. We observed that 83% (45/54) of the cities had at least one seropositive animal (S1 Table).

## Discussion

During the study, we observed *C*. *burnetii* positivity in beef cattle sent to slaughter using serological and molecular methods. Cattle exposure to the bacterium could be verified through IFA in 360 of the 1515 samples (23.8%), which indicates a past or current infection. The presence of the pathogen in the bloodstream of cattles at the time of slaughter was confirmed using qPCR in 12.2% (44/360) of the seropositive animals, and this finding is suggestive of bacteremia.

In several countries, the use of serological diagnoses in slaughterhouses is the primary method of zoonotic disease surveillance. The efficiency of this approach relies on a traceability system, which is useful for detecting and monitoring pathogens in regions with relatively high prevalence [55]. Sampling in abattoirs has some advantages when the prevalence of a disease is unknown, such as the sampling of a large number of animals from different cities, regions, and producers in a short time. However, some pathogens have specific infection characteristics, such as a preference for sex, age, or type of production (extensive versus intensive) [56]. Therefore, the results obtained from samples taken at slaughterhouses can be adjusted to estimate the prevalence in the living population, if necessary; for example, when differences in prevalence are expected in different age or sex cohorts. The slaughter animal population is mainly composed of healthy males. Thus, our results estimate *C*. *burnetii* infections in beef cattle, rather than the prevalence of coxiellosis disease, since most animals are asymptomatic [1, 57, 58]. A recent study conducted in small ruminants in slaughterhouses suggested a lack of correlation between sex and Q fever positivity in serodiagnoses, and similar prevalence rates were observed among males and females [59]. However, the evaluation of females from herds that experience abortions due to Q fever can lead to higher seroprevalence rates, as observed in northeast Brazil, where 55.1% (172/312) of dairy goats were positive for *C*. *burnetii* [15].

The median prevalence reported worldwide for cattle is 37.7% [10]. Although lower than the reported average, the true prevalence of 20.0% that was found in the IFA serological analysis is considered high. Our results are similar to those of a previous study of slaughterhouses in Brazil, where the prevalence assessed using the complement fixation test was 29% [14]. The serology diagnosis outcome can influence the antigen used [60]. Notably, studies that compared antigens produced from the Nine Mile strain, a bovine strain, and a ruminant strain reported better efficiency in sensibility and specificity bovine and ruminant strains [11, 61]. Additionally, phase II LPS is an immunodominant antigen that is inserted in phase I LPS; therefore, antibodies against phase II are always present during the infection and have a higher prevalence in epidemiological studies [18, 62]. In our study, we used an in-house antigen produced from a *C*. *burnetii* strain that was isolated from ticks in Argentina. Since it was a low-passage Vero cell culture strain, it is possible that it is mainly composed of phase I cells. Therefore, the estimated prevalence can be even higher because antibodies against phase II might be underestimated. Furthermore, the use of a tick strain from Argentina can also impact the study outcome. Serological evidence of *C*. *burnetii* can reflect a recent or past exposure to the pathogen; therefore, it cannot infer an actual hazard for slaughterhouse workers. However, qPCR detection in 12.2% (44/360) of the seropositive samples revealed presence of the pathogen at the moment of slaughter.

In humans, the diagnosis of acute Q fever using qPCR in sera is indispensable for diagnosing bacteremia. The use of molecular detection in sera was previously deployed to identify the source of a human infection in Japan, where two patients, two dogs, and three cats were positive by nested PCR. Moreover, the investigators were able to isolate *C*. *burnetii* from the serum samples, which demonstrates the presence of viable bacteria in the bloodstream of the animals [21]. Recently, *C*. *burnetiid* was isolated from the serum of a patient with acute Q fever in Australia using cell culture [63], and this finding corroborates the presence of viable bacteria in the sera and blood of infected hosts. Our results suggest that bacteremia is present in a significant proportion of tested animals and most likely represents active infections. Due to the lack of data that correlates bacteria detection in sera with clinical disease or shedding in animals, we can only wonder if bacteremia is associated with primary infection exclusively or with chronic phases that result in an active infection.

The molecular detection of *C*. *burnetii* in the bloodstream of cattle was confirmed by phylogenetic analysis of *IS1111a* sequences. All seven sequences that were investigated in our study were clustered together, along the 16 *IS1111a* of *C*. *burnetii* strain sequences that were used in the comparison (S1 Fig). Moreover, the *IS1111*-like CLB sequence appeared in a different branch, which demonstrates the specificity of the Trans 3 and Trans 4 primers that were used in this study. *Coxiella*-like bacteria have a symbiont relationship with ticks [64] and are widespread in these arthropods, with a 100% prevalence in some species [8]. This can lead to the transmission of CLB to animals, as reported in birds [65, 66] and horses [67]. Additionally, the existence of an *IS1111*-like CLB with 90% identity to *IS1111* has been found in *C*. *burnetii* [50] and can impact Q fever diagnosis if these species are misidentified.

The lack of discrimination between *C*. *burnetii* strains using the *IS1111a* marker is probably due to the small amplicon size (243 bp) and its location in a conserved region of the gene. Notably, studies that used Trans 1 and Trans 2 primers, which produce a 600 bp amplicon, identified differences between *C*. *burnetii* strains derived from different hosts and countries [68, 69]. Nevertheless, recent multi spacer typing (MST) and multi-locus variable tandem repeat analysis (MLVA) investigations in Brazil have identified evidence of unique genotypes [17], and this emphasizes the need of epidemiological and virulence studies that aim to better understand the disease in this region. MST-74, detected in Brazil, belongs to the same phylogenetic branch as MST 20 and MST 61 [16], which places the strains circulating in Brazil in genomic group III of Hendrix et al. [70–72]. In a recent virulence study, it was shown that strains belonging to genomic groups I and III were more virulent, with increased pathogenic potential compared to *C*. *burnetii* strains from other genomic groups [73].

The main route of Q fever transmission is through the inhalation of infectious particles. Quantification of *C*. *burnetii* in the bloodstream through qPCR showed a concentration that ranged from 10 to 814 bacteria / mL (average of 144 cells / mL). As *C*. *burnetii* is an intracellular pathogen [3], there are more bacteria per milliliter in blood than in the sera of slaughtered cattle. During bleeding, cattle eliminate approximately 4.5 liters of blood, which can result in the dissemination of approximately 6x10^4^ bacteria, at the abovementioned concentration. Thus, the results presented here evidentiate the risk of infection for slaughterhouse workers due to aerosol formation during slaughter procedures, such as bleeding and sawing of half-carcasses, among others [74]. These facts emphasize the risk of acquiring Q fever due to the contact with blood from infected animals, a possibility that increases inside abattoirs due to the formation of aerosols from slaughter procedures and the main infective respiratory route of *C*. *burnetii* in humans [74, 75].

The lack of human Q fever surveillance in Brazil, in addition to the high animal prevalence reported here and by other authors in Brazil [13–15], suggests that Q fever must be a neglected disease due to misdiagnosis in Brazil. Active infection also represents a threat for people who live in areas that surround livestock once asymptomatic and symptomatic animals shed bacteria into the environment, leading to possible *C*. *burnetii* dissemination to urban areas, through the wind over long distances [5, 6]. Additionally, the possibility of transmission through consumption of cattle milk and dairy products should not be neglected, as the presence of *C*. *burnetii* has been evaluated in such products in Brazil [16, 76].

## Conclusion

The high prevalence of *C*. *burnetii* infection in cattle sent to slaughter and the wide dispersion observed in the density maps indicates that the pathogen is widely prevalent in herds of the State of São Paulo and is not restricted to a single region. These results indicate a risk for public health, since *C*. *burnetii* is an airborne pathogen that can be spread by wind over long distances and cause Q fever outbreaks in cities close to livestock production. In addition, the occupational hazard for farmers, veterinarians, and especially, abattoir workers, has to be highlighted due to the potential accumulation of *C*. *burnetii* contamination in their environments. Surveillance programs for humans and animals should be implemented in Brazil to monitor cases of Q fever and establish preventive and control measures.

## Supporting information

S1 Fig Phylogenetic tree based on the *IS1111a* sequence of *Coxiella burnetii* strains and *IS1111-like* sequence derived from a Coxiella-like bacteria (CLB). (TIF)

S1 Table Distribution of samples collected in the slaughterhouses according to the city of origin of the herd. (XLSX)
