# Supplementary figures and images for: Coxiella burnetii in slaughterhouses in Brazil: A public health concern
Source: PLoS One. 2020 Oct 30;15(10):e0241246. doi: 10.1371/journal.pone.0241246 (PMC7598456; doi:10.1371/journal.pone.0241246)

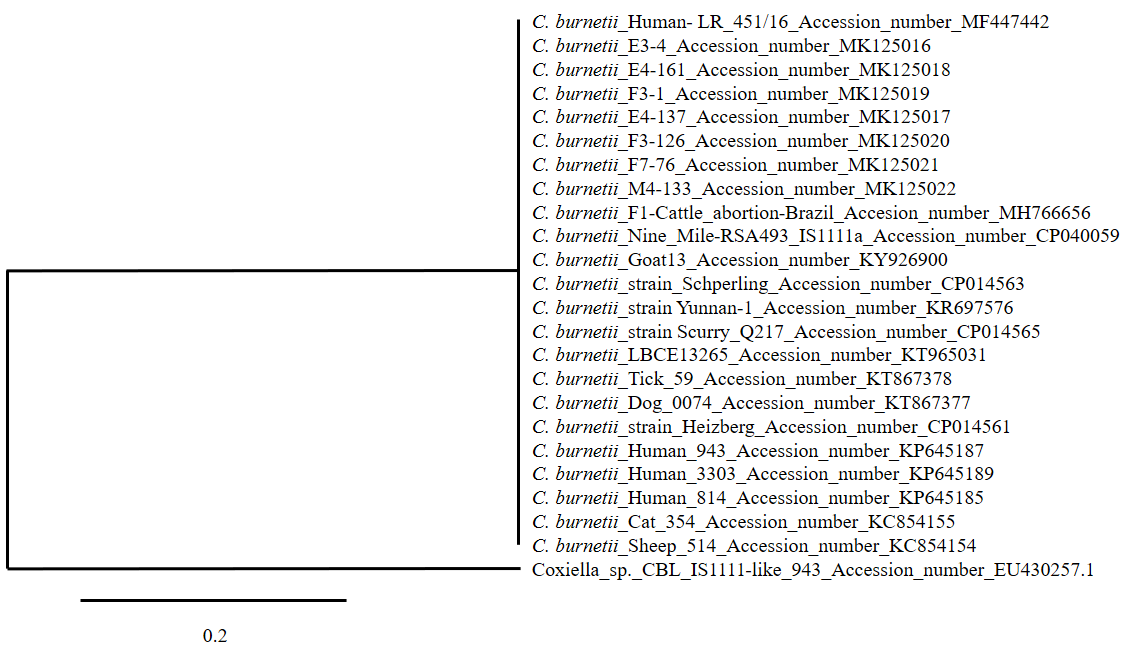

Supplement: S1 Fig — (TIF) [file pone.0241246.s001.tif]
